# Acute and subacute toxicity evaluation of hydroalcoholic extract from the stem bark of Bois Bande (*Parinari campestris* Aubl.1772) in rats

**DOI:** 10.1186/s40360-021-00522-w

**Published:** 2021-09-25

**Authors:** Venkatesan Sundaram, Stephanie Mohammed, M. R. Srinivasan, Jenelle Johnson, Rod Suepaul, Indira Pargass, Chernell John, Danesha Ramdhanie, Shiann Lallack, Esther Daniel, Lester Gilkes

**Affiliations:** 1 Department of Basic Veterinary Sciences, School of Veterinary Medicine, Faculty of Medical Sciences, The University of the West Indies, St. Augustine, Trinidad and Tobago; 2 Biochemistry Unit, Department of Preclinical Sciences, Faculty of Medical Sciences, Faculty of Science and Technology, The University of the West Indies, St. Augustine, Trinidad and Tobago; 3 Department of Veterinary Pharmacology and Toxicology, Madras Veterinary College, Tamilnadu Veterinary and Animal Sciences University, Tamil Nadu Chennai, India; 4 Department of Clinical Veterinary Sciences, School of Veterinary Medicine, Faculty of Medical Sciences, The University of the West Indies, St. Augustine, Trinidad and Tobago

**Keywords:** Bois Bande, *Parinari campestris*, Acute toxicity, Subacute toxicity

## Abstract

**Introduction:**

The bark of Bois Bande (*Parinari campestris*) is a popular aphrodisiac in the Caribbean that has been traditionally used for many years to restore sexual vitality, increase sperm count, and treat erectile dysfunction, without valid scientific data. Acute and 28-day subacute toxicity studies were conducted to evaluate the safety of the hydroalcoholic extract of *P.campestris* bark and to find a safe dose for human use in conventional medicine.

**Methods:**

The acute toxicity study used a single oral dose of *P.campestris* extract at four separate doses, 5, 50, 300, and 2,000 mg/kg, and was seen for 14 days, while the subacute toxicity study used a daily oral dose of *P.campestris* extract at 3 different doses, 100, 300, and 1000 mg/kg/day for 28 days.

**Results:**

The LD_50_ of *P.campestris* extract was found to be greater than 2000 mg/kg in the acute toxicity study. *P.campestris* extract did not show toxicity at 1000 mg/kg/day in subacute toxicity trial; NOAEL was 1000 mg/kg/day in rats. However, the body weight was increased in males.

**Conclusion:**

In conclusion, 1000 mg/kg *P.campestris* extract can be considered safe and non-toxic in males.

**Supplementary Information:**

The online version contains supplementary material available at 10.1186/s40360-021-00522-w.

## Introduction

Approximately 31–52 % of the world’s male population has met sexual issues linked to ejaculation disorders, erectile dysfunction, and suppressed sexual desire [1, 2]. The World Health Organization has emphasized that not only the lack of disease, dysfunction, or sickness but also the satisfaction and positive role of sexual health are critical for sexual health [3]. The enhancement of sexual activity is believed to improve the satisfaction and self-esteem of human relationships [4]. Consequently, the quest for natural aphrodisiacs that arouses sexual desire to enhance pleasure and performance has increased. This pursuit of happiness made many herb-based natural aphrodisiacs popular all over the world in recent years due to their low cost, easy availability, and promotion as having “no side effects.” However, most of the products were not confirmed scientifically for their safety, side effects, and efficacy.

Natural products are widely acknowledged as safer in the health maintenance of developed countries and commonly used as self-medication even though no specific toxicological profile was provided [5]. The toxicity of herbal compounds has been documented in different studies [6, 7]. The homeostasis and defense system of the human body was affected by delayed elimination of toxic chemicals in the natural products as well as long-term exposure of less harmful substances even at low doses [8]. So, the toxicity evaluation of the natural products becomes more important to ensure the safety of human health as the usage of these products increasing every day.

*Parinari campestris* Aubl.1772, a West Indian tropical tree plant belonging to the *Chrysobalanaceae* family, is commonly known as Bois bande or Bwa bandé along with other tree plants *Richeria grandis* and *Roupala montana.* The bark of these trees is highly reputed for their aphrodisiac properties in the Caribbean region. The *P.campestris* bark is used to restore sexual vitality, increase sperm count and counteract erectile dysfunction traditionally for many years [9]. In recent years, Bois bande has enjoyed great commercial popularity all over the world due to folklore, the internet, and tourism. However, there is no scientific data on its toxicity and side effects except an anecdotal warning that consumption at high doses may cause priapism, a persistent and painful erection lasting more than six hours with the loss of energy. Therefore, a toxicological evaluation of hydroalcoholic extract of *Parinari campestris* stem bark was done in rats, a primary predictive model for human effects in toxicity testing to assess its safety and finding a safe dose for the use of humans in conventional medicine.

## Materials and methods

### Plant collection and extraction

The fresh stem bark of *P.campestris* was collected from Mt. Harris Forest (10º30’38” N 61º6’23” W), Trinidad and Tobago with approval from the Forestry Division of the Ministry of Agriculture, Land and Fisheries, Trinidad, and Tobago. The plant was identified by technical staff at the National Herbarium of Trinidad and Tobago, Department of Life Sciences, University of the West Indies, St. Augustine, Trinidad, and Tobago, and voucher specimen (No. TRIN 50,648) was preserved. The samples were dried for 10 days and powdered. The powdered sample was extracted with 70 % ethanol at room temperature (23 °C) for 7 days with a material to solvent ratio of 1:4 (w: v) and vacuum filtered by a Buchner funnel. The process was repeated three times with fresh solvent each time and the combined filtrate was allowed to dry in a hot air oven at 37–40 °C and a dry hydroalcoholic extract was obtained with a yield percentage of 3.2 w/w %.

### Animal Care and Husbandry

In the present study, sixty-three (63) healthy young adult (8–10 weeks old) Sprague Dawley rats of either sex (15 males for acute toxicity test and 24 males and 24 females for subacute toxicity test) with a weight range of 195-257 g were used. The animals were purchased from the Lab Animal Facility of the School of Veterinary Medicine, The University of the West Indies, St. Augustine, Trinidad, and Tobago. The rats were divided randomly by random numbers generated from Microsoft excel using = RAND () into groups according to the OECD guidelines (no.423 and no.407) and each group was housed in its cage in a dedicated experimental room at the School of Veterinary Medicine with a temperature of 22 ± 3 °C and relative humidity of 50–60 %, as well as a 12-hour light/dark artificial light period. The animals were fed regular pellet feed and given free access to water. Before the experiment, all the animals were given a 7-day acclimatization period in the laboratory. The Campus Research Ethics Committee, The University of the West Indies, St. Augustine, Trinidad, and Tobago (No. CREC-SA.0072/11/2019) approved all animal procedures as per the National Institutes of Health’s Guide for the Care and Use of Laboratory Animals [10].

### Acute toxicity study

Research guideline no.423 of the Organization for Economic Cooperation and Development (OECD) was followed to conduct the acute oral toxicity analysis [11]. The study was performed on male rats because males were the intended sex in this research. A total of 15 male rats weighing 200 to 250 g were divided into five experimental groups of three rats each (control, 5, 50, 300, and 2000 mg/kg groups). The limit test dose of 2000 mg/kg was selected as per the OECD Guidelines since no previous information was available on the toxicity of the *P.campestris* extract. The doses were prepared in sodium carboxymethyl cellulose (0.25 %) solution to form a uniform suspension and administered by oral gavage. The control group was administered with sodium carboxymethyl cellulose (0.25 %) solution whereas the other four groups received single oral doses of *P.campestris* extract at 5, 50, 300, and 2000 mg/kg daily at 9 am. On the first day after the gavage, all animals were monitored for mortality and general behavioral changes for 30 min, 2 h, 4 h, 6 h, 10 h, and 24 h, and then for a total of 14 days daily once. The median lethal dose (LD_50_) was estimated as per OECD guideline No.423 [11]. All the animals were euthanized on the 15th day by intraperitoneal injection of pentobarbital sodium (120 mg/kg) [12], and post mortem examination was carried out to investigate the gross pathology.

### 28-day subacute toxicity study

The OECD Guideline no.407 was used to conduct the 28-day subacute toxicity test [13]. Forty-eight rats were divided into four groups at random, each with 12 animals (6 males and 6 females). The LD_50_ was found to be greater than 2000 mg/kg as per the acute toxicity study. The control group was administered with carboxymethylcellulose (0.25 %) whereas the other three groups were received *P.campestris* extract daily by oral gavage at 100, 300, and 1000 mg/kg for 28 days daily at 9 am based on the estimated LD_50_ of the *P.campestris* extract. During treatment, irregular behavior, adverse clinical symptoms, and mortality were observed daily. The body weight, feed intake, and water consumption were recorded weekly. After an overnight fast, the animals were weighed and sedated with an intraperitoneal injection of ketamine hydrochloride (80 mg/kg). Following the sedation, the rats were anesthetized with intraperitoneal administration of pentobarbital sodium (40 mg/kg) [12], and blood samples were collected by an intracardiac puncture for hematological and biochemical analysis. All the animals were euthanized after the blood collection by intraperitoneal injection of pentobarbital sodium (120 mg/kg) [12]. The organs such as liver, kidney, spleen, heart, brain, lungs, testes, and ovaries were collected and weighed to calculate the relative organ weights (dividing each animal’s organ weight by their body weight). The samples from the tissues were collected and fixed in 10 % buffered formalin for histopathological examination.

#### Hematological and Biochemical analysis

The blood was collected in vacutainer tubes coated with Ethylenediaminetetraacetic acid (EDTA) for the hematological analysis. The blood parameters like White Blood Cell Counts (WBCs), Red Blood Cell Counts (RBCs), Hematocrit (HCT), Hemoglobin (Hb), Mean Corpuscular Volume (MCV), Mean Corpuscular Hemoglobin (MCH), Mean Cell Hemoglobin Concentration (MCHC), Red Blood Cell Distribution Width (RDW), Reticulocytes (REL), Platelets (PLT), and Mean Platelet Volume (MPV) were estimated by using an automatic hematology analyzer (ProCyte Dx™, Idexx Laboratories, Maine, USA).

Regarding biochemical analysis, solidified blood samples in non-EDTA coated tubes were centrifuged by a tabletop centrifuge (TJ-6, Beckman Coulter Inc., Brea, USA) at 1000 g at room temperature for 10 min to get sera for analysis. The serum biochemical parameters such as Serum Na, Serum Potassium (K), Sodium Potassium (Na: K) ratio, Serum Chloride (Cl), Urea, Creatinine, Total Protein (TP), Albumin, Globulin, Albumin: Globulin (A: G) ratio, Glucose, and Cholesterol, Alkaline phosphatase (ALP), Alanine aminotransferase (ALT), Aspartate aminotransferase (AST), were analyzed by chemistry analyzer (BS 200, Mindray Medical International Company, Shenzhen, China).

#### Histopathological analysis

Tissue specimens were fixed in 10 % buffered formalin and processed by routine histological processing. The histological sections of 4 μm thickness were cut by using a rotary microtome (Finesse ME, Thermo Scientific Fisher Company, Waltham, USA) and stained with Haematoxylin and Eosin (H&E). The sections were examined with the aid of an Olympus BX51 system microscope with a digital camera (Olympus Corporation, Tokyo, Japan).

### Statistical analysis

All data were analyzed blindly and expressed as mean ± SEM. Individual group data was checked for equality of variance by the Brown-Forsythe test. If the variance of the treatment groups was homogenous, then the parametric test, one-way ANOVA followed by the post-hoc Dunnett’s multiple comparison test to compare the treatment group with the control group. If there was significant inequality of variance occurs, then the non-parametric test, equivalent to one-way ANOVA, the Kruskal-Wallis test was done followed by the post-hoc Dunnett’s multiple comparison test to compare the treatment group with the control group. *p* < 0.05 was considered significant. All statistical analysis was performed with GraphPad Prism (Version 9.0) software (GraphPad Software Inc, CA, USA).

## Results

### Acute toxicity study

The acute toxicity study revealed no mortality, morbidity, unusual behavior, and adverse clinical signs at all the tested single oral doses (5, 50, 300, and 2000 mg/ kg). The post mortem examination also did not show any gross pathological changes in all the animals studied. As a result, the extract’s LD50 in rats was estimated to be greater than 2000 mg/kg.

### 28-day subacute toxicity study

#### Behavior, Bodyweight, feed, and water intake

**Fig. 1:**
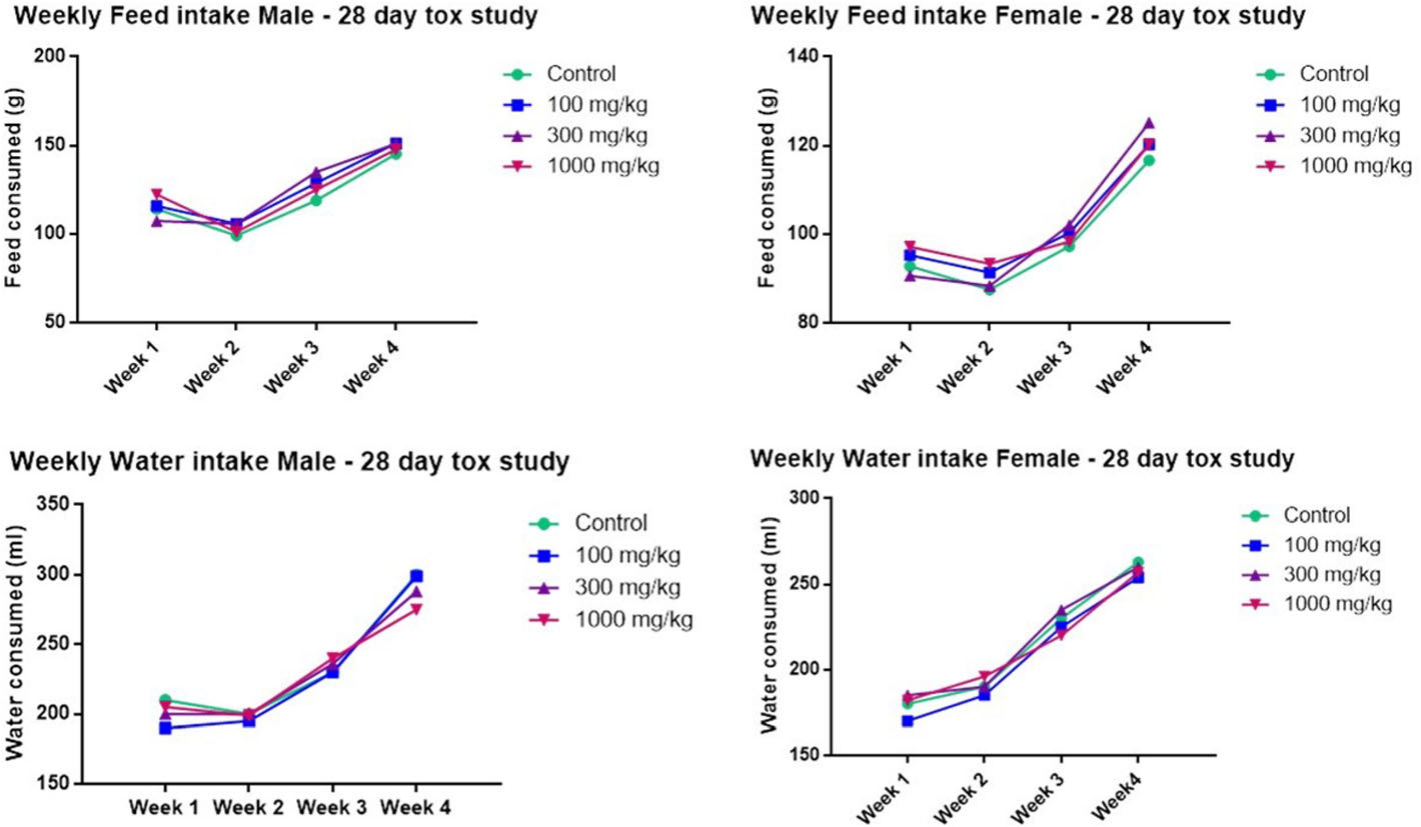
The feed and water intake data of rats in 28-day subacute toxicity (n = 6)

The 28-day subacute toxicity study did not exhibit any mortality, morbidity, unusual behavior, and adverse clinical signs in all animals studied. The body weight increased gradually without significance from week 1–4 in all the animals of both sexes (Table 1). In males, a significant increase was noticed in the 100 mg/kg group on the third week, 100 and 300 mg /kg group on the 4th week, and 100, 300, and 1000 mg/kg groups (p < 0.05) on the day of sacrifice which was on 29th day (fasted body weight). In females, no statistically significant changes in the body weight were noticed except a decrease in the 1000 mg/kg group in fasted body weight (*p* < 0.05). The total body weight gain was significantly higher in the 100 mg/kg treated group in males and lower in the 1000 mg/kg treated group in females (p < 0.05). No significant treatment-related change was observed in the feed and water intake of all the animals (Fig. 1).

**Table.1:** Summary of weekly body weight changes (g) of rats in 28-day subacute toxicity study (*n* = 6)

	Males				Females			
Weeks	Control	100 mg/kg	300 mg/kg	1000 mg/kg	Control	100 mg/kg	300 mg/kg	1000 mg/kg
Week 1	209.08 ± 25.22	241.18 ± 7.70	244.36 ± 6.06	257.35 ± 7.90	195.79 ± 5.02	192.03 ± 7.81	210.46 ± 5.24	203.38 ± 3.61
Week 2	246.70 ± 27.37	295.43 ± 7.31	294.03 ± 8.83	268.45 ± 12.73	226.06 ± 3.86	221.82 ± 6.71	238.24 ± 6.75	216.88 ± 7.04
Week 3	272.01 ± 27.66	337.37 ± 7.08*	331.38 ± 7.76	296.94 ± 7.09	247.47 ± 4.23	258.98 ± 17.80	248.24 ± 6.75	227.38 ± 5.29
Week 4	289.45 ± 29.13	368.89 ± 9.62*	364.33 ± 7.17*	338.00 ± 7.86	266.61 ± 5.42	259.98 ± 6.76	267.98 ± 7.39	245.04 ± 5.44
Fasted body weight	293.83 ± 29.50	374.89 ± 10.68*	367.83 ± 9.38*	351.17 ± 6.70*	269.50 ± 4.66	256.50 ± 6.05	264.83 ± 5.87	249.00 ± 4.38*
Body weight gain (Week 4–Week 1)	102.90 ± 14.66	127.70 ± 9.95*	120.00 ± 10.53	80.66 ± 8.00	70.82 ± 6.55	67.95 ± 3.35	57.53 ± 7.60	41.66 ± 8.50*

Note. Data was stated as Mean ± Standard Error of the Mean (SEM). *Significantly different from the control group, *p* < 0.05.

#### Gross pathology and Relative organ weights

The post mortem examination did not show any abnormal changes or lesions in the internal organs of all the groups. No significant difference was noticed in the mean relative organ weights between control and treated groups (*p* > 0.05) except ovaries (Table 2). The relative ovaries weight was significantly increased (p > 0.05) in 100 and 1000 mg /kg groups.

**Table.2:** Summary of relative organ weights (g) in 28-day subacute toxicity study (n = 12)

Relative organ weights (g)	Control	100 mg/kg	300 mg/kg	1000 mg/kg
Liver	0.036 ± 0.001	0.039 ± 0.001	0.039 ± 0.001	0.035 ± 0.001
Kidneys	0.009 ± 0.000	0.009 ± 0.001	0.010 ± 0.001	0.008 ± 0.000
Spleen	0.002 ± 0.000	0.003 ± 0.001	0.003 ± 0.001	0.002 ± 0.000
Heart	0.004 ± 0.000	0.004 ± 0.001	0.005 ± 0.001	0.003 ± 0.000
Lungs	0.007 ± 0.000	0.006 ± 0.001	0.007 ± 0.001	0.006 ± 0.001
Brain	0.006 ± 0.000	0.006 ± 0.001	0.007 ± 0.001	0.006 ± 0.000
Testes	0.009 ± 0.001	0.009 ± 0.001	0.010 ± 0.001	0.008 ± 0.000
Ovaries	0.0004 ± 0.000	0.002 ± 0.000*	0.002 ± 0.001	0.001 ± 0.000*

Note. Data was stated as Mean ± Standard Error of the Mean (SEM). *Significantly different from the control group, *p* < 0.05

#### Hematological Analysis

The values of all the hematological parameters studied in all the animals were within the reference range for rats as shown in Table 3. The values of control and *P.campestris* extract-treated rats did not differ significantly (*p* > 0.05) except for a significant increase in PLT (*p* < 0.05) in the 300 mg/kg group (Table 3).

**Table.3:** Summary of the hematological parameters of the rats in subacute 28-day subacute toxicity study (*n* = 12)

Parameters	Control	100 mg/kg	300 mg/kg	1000 mg/kg
WBC (10^9^/L)	8.42 ± 0.75	7.75 ± 0.52	8.70 ± 0.54	7.44 ± 0.58
RBC (10^12^/L)	7.76 ± 0.14	8.09 ± 0.13	8.25 ± 0.12	7.89 ± 0.22
HCT (L/L)	0.41 ± 0.01	0.43 ± 0.03	0.43 ± 0.01	0.43 ± 0.01
Hb (g/L)	147.00 ± 1.90	152.70 ± 2.30	152.80 ± 1.70	151.3 ± 2.02
MCV (fL)	53.06 ± 0.20	53.19 ± 0.33	52.05 ± 0.41	54.42 ± 0.86
MCH (pg)	19.06 ± 0.14	18.88 ± 0.08	18.55 ± 0.14	19.30 ± 0.47
MCHC (g/L)	349.4 ± 10.77	354.30 ± 2.18	356.60 ± 2.15	354.4 ± 3.44
RDW (%)	21.64 ± 0.82	20.30 ± 0.24	21.19 ± 0.36	21.52 ± 0.29
Reticulocytes (REL) (10^9^/L)	4.30 ± 0.27	3.54 ± 0.21	3.47 ± 0.22	4.52 ± 0.28
Platelets (PLT) (10^9^/L)	743.00 ± 55.25	942.20 ± 50.12	987.50 ± 61.24*	731.40 ± 92.78
MPV (fL)	8.79 ± 0.18	9.08 ± 0.11	9.06 ± 0.18	9.09 ± 0.09

*Note.* Data was stated as Mean ± Standard Error of the Mean (SEM). *Significantly different from the control group, *p* < 0.05

#### Serum Biochemical Analysis

All the biochemical parameters studied in the treated groups were within the reference range for rats. The values of control and *P.campestris* extract-treated rats did not differ significantly (p > 0.05) except for a significant decrease in AST (p < 0.05) in the 300 mg/kg group (Table 4).

**Table.4:** Summary of the biochemical parameters of the rats in 28-day subacute toxicity study (*n* = 12)

Parameters	Control	100 mg/kg	300 mg/kg	1000 mg/kg
Serum Na (mmol/L)	142.70 ± 0.49	142.10 ± 0.31	141.90 ± 0.42	142.20 ± 0.42
Serum K (mmol/L)	4.78 ± 0.19	5.13 ± 0.40	4.55 ± 0.14	5.12 ± 0.22
Sodium: Potassium ratio	30.48 ± 1.19	29.20 ± 1.72	31.84 ± 0.99	28.35 ± 1.22
Serum Cl (mmol/L)	106.00 ± 0.66	107.40 ± 0.56	105.80 ± 0.33	106.30 ± 0.37
Urea ((mmol/L)	6.66 ± 0.35	6.18 ± 0.22	6.36 ± 0.24	6.18 ± 0.11
Creatinine (µmol/L)	24.83 ± 1.19	26.07 ± 1.73	25.21 ± 1.14	22.38 ± 0.85
Total Protein (g/dL)	70.75 ± 0.52	72.33 ± 1.03	71.36 ± 0.87	72.50 ± 0.82
Albumin (g/L)	34.50 ± 0.66	35.45 ± 0.49	35.00 ± 0.36	36.42 ± 0.66
Globulin (g/L)	36.25 ± 0.87	36.67 ± 0.77	36.36 ± 0.90	35.82 ± 1.01
Albumin: Globulin ratio	0.96 ± 0.04	0.96 ± 0.02	0.97 ± 0.03	1.03 ± 0.05
Glucose (mmol/L)	6.27 ± 0.28	7.06 ± 0.33	6.92 ± 0.30	7.01 ± 0.59
Cholesterol (mmol/L)	1.48 ± 0.07	1.59 ± 0.09	1.61 ± 0.07	1.36 ± 0.10
ALP (U/L)	220.00 ± 48.98	167.50 ± 12.86	153.20 ± 10.17	170.50 ± 9.61
ALT(U/L)	60.36 ± 4.45	69.36 ± 8.76	60.73 ± 4.75	69.50 ± 6.45
AST (U/L)	172.80 ± 12.07	132.30 ± 17.92	99.45 ± 6.78*	177.20 ± 33.45
*Note.* Data was stated as Mean ± Standard Error of the Mean (SEM). *Significantly different from the control group, p < 0.05.				

#### Histopathological analysis

**Fig. 2:**
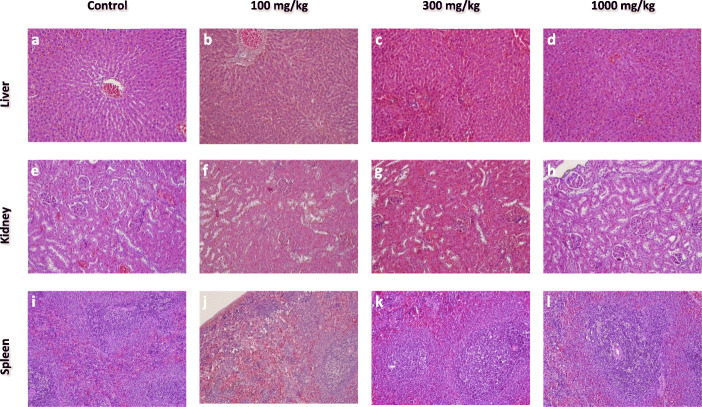
The histopathology of liver, kidney, and spleen of the rats in 28-day subacute toxicity study. The liver (**a**-**d**) with typical hepatic cords with sinusoids with the central vein, the kidney (**e**-**h**) showed normal renal corpuscles, tubules, and collecting ducts, the spleen (**i**-**l**) with regular red and white pulp architecture (H&E x 200).

**Fig. 3:**
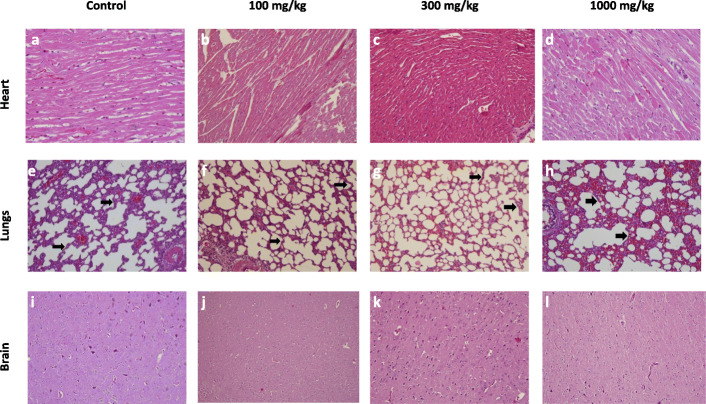
The histopathology of the heart, lungs, and brain of the rats in 28-day subacute toxicity study. The heart (**a**-**d**) showed normal myocardium. The lungs (**e**-**h**) showed mild congestion and thickened alveolar walls (arrows) in all the groups including the control group. The brain (**i**-**l**) showed normal neuronal architecture with glial cells (H&E x200)

**Fig. 4:**
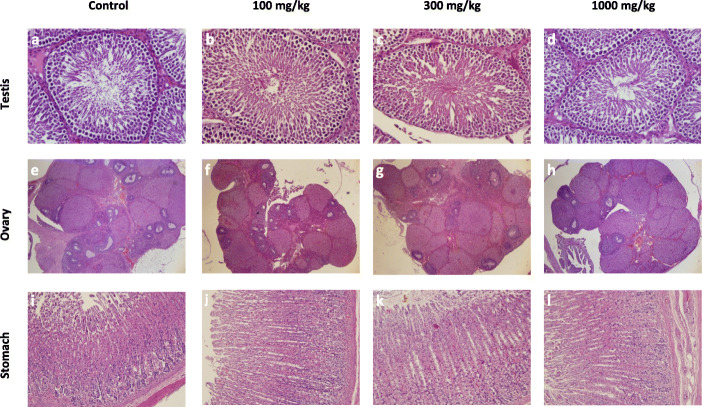
The histopathology of the testes, ovary, and stomach of the rats in 28-day subacute toxicity study. The testes (a-d) showed regular seminiferous tubules and interstitial cells (H&E x400), the ovary (**e**-**h**) showed normal follicular development, and interstitium (H&E x40), the stomach (**i**-**l**) showed normal gastric mucosa and glands (H&E x200)

**Fig. 5:**
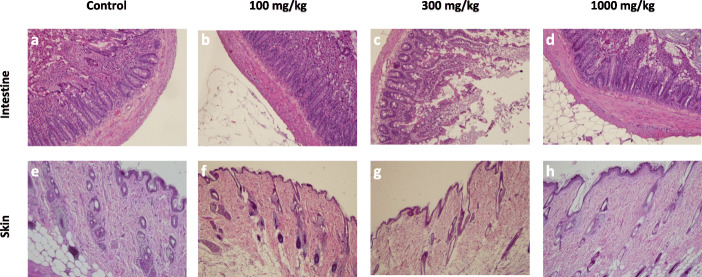
The histopathology of the intestines and skin of the rats in 28-day subacute toxicity study. The intestine (**a**-**d**) showed typical mucosa with villi lined by enterocytes and goblet cells and the skin (**e**-**h**) showed the intact epidermis and dermis with normal sebaceous, sweat glands, and hair follicles (H&E 200).

The histopathological analysis of major organs like liver, kidney, testes, brain, heart, lungs, testes, spleen, ovary, stomach, intestine, and skin of all the groups (control, 100, 300, and 1000 mg/kg) was done. The histopathological evaluation showed normal architecture (Figs.2, 3, 4, 5) comparable to the controls and there were no dose-dependent changes. The liver exhibited normal hepatic cords and sinusoids with typical hepatocytes and no signs of apoptosis (Fig.2 a-d). The kidney revealed regular renal corpuscles, tubules, and collecting ducts with normal interstitial tissue (Fig.2 e-h). The spleen showed normal red and white pulps (Fig.2 i-l). The heart myocardium revealed intact muscle bundles with normal muscle cells with typical vasculature (Fig.3 a-d). The regular neuronal architecture with glial cells was seen in the brain (Fig.3 e-h). Mild alveolar tissue congestion and alveolar thickening were noticed in all the groups including control (Fig.3 i-l). The testes showed the regular seminiferous tubules with progressive spermatogenesis and normal interstitial cells (Fig.4 a-d). The ovary showed normal follicular development and interstitium (Fig.4 e-h). The gastric mucosa, glands, and musculature were typical with the normal chief and parietal cells were found in the stomach (Fig.4 i-l). The intestinal tissue showed typical mucosa with villi lined by enterocytes and goblet cells (Fig.5 a-d). The skin showed intact epidermis and dermis with normal sebaceous and sweat glands with healthy hair follicles (Fig. 5 e-h).

## Discussion

The *P.campestris* is used as a traditional medicine in curing erectile dysfunction without any scientific data on its toxicity profile to date. Since toxicological screening is a crucial tool to evaluate the safety of drugs or plants [14], acute and sub-acute oral toxicity studies of *P.campestris* extract were conducted. The rats are one of the most important animal models in toxicology. Hematological, respiratory, and cardiovascular adverse effects have the greatest cumulative comparability of toxicity in animals and humans [15]. However, the toxicity observed in animals is poorly correlated with certain adverse effects like hypersensitivity and idiosyncratic reactions in humans. Besides, side effects such as headache, stomach pain, dizziness, and visual hallucinations are also difficult to detect in animals. Moreover, it is difficult to extrapolate certain adverse effects between the species due to the pharmacokinetic variations between the species. However, rats are the primary predictive models for human effects in toxicity assessments [16] and the study was done on rats as per OECD guidelines [11, 13].

The phytochemical screening is usually done for the aphrodisiac plants to detect the phytoconstituents like carbohydrates, alkaloids, proteins, amino acids, tannins, phenolics, saponins, flavonoids, triterpenoids, steroids, glycosides, fixed oils, gums, and mucilages. So far no studies were conducted on *P.campestris* except a study that identified a new kaurene diterpene dimer [17]. However, the Parinari species showed a predominance of flavonoids glycosides based on myricetin, quercetin, and kaempferol [18]. The present study focuses only on the toxicological evaluation and the second phase of this ongoing research is aimed to carry out the phytochemical analysis and evaluation of the aphrodisiac potential of *P.campestris*.

The acute toxicity tests did not exhibit any mortality, morbidity, unusual behavior, and adverse clinical signs in all the animals. The *P.campestris* extract can therefore be considered non-toxic up to 2000 mg/kg single oral administration and can be classified as a Class 4 drug as per the acute toxicity classification criteria for substances [13, 19].

The *P.campestris* stem bark is consumed as an herbal brew (approximately 20–50 g of bark infused into alcohol for a week) or tea (a spoon of stem bark powder, roughly 5–8 g, added to one liter of boiling water). It can be interpreted that the LD_50_ dose was much higher than the amount consumed in conventional medicine by considering the yield percentage (3.2 % w/w) and the assumed LD_50_ (2000 mg/kg) of the *P.campestris* extract in the present study. It should also be remembered that, relative to humans, rodents are more vulnerable to oral toxicity [20].

Toxicological assessments provide a dose-response relationship on potential health risks after repeated administrations. Thus, three different doses (100, 300, and 1000 mg/kg/day) were tested in both sexes for 28 days in a subacute oral toxicity study. The non-significant increase in body weight every week indicated that the feed and water intake was proportionate to the normal growth and physiology. However, a significant increase in body weight in the 100 mg /kg group on week 3 and the 100 and 300 mg/kg groups on week 4 and in the 100, 300, and 1000 mg/kg groups on the day of sacrifice (fasted body weight) in males indicated the treatment-related effect. This increase was not considered as an adverse effect since it could be due to body fat accumulation [21] or muscular development during the treatment due to the possible aphrodisiac effect. In the females, the treatment-related effect was seen as a decrease in body weight and body weight gain in the high dose (1000 mg/kg) group which could be a dose-dependent effect. It needs further investigation as body weight decrease was seen only in the 1000 mg/kg group So, the above results indicate that *P.campestris* extract increases the weight in males.

The detrimental effect of the test drug on metabolism is demonstrated by any major changes in food and water consumption [22]. No treatment-related effect was noticed in the feed ad water intake in the present study. Therefore, the *P.campestris* extract did not cause any change in appetite or thirst and alter the metabolic system on long-term administration.

The relative organ weight is considered one of the primary indicators of organ toxicity. The relative organ weights did not differ significantly except for the ovaries. The increase in relative ovaries weights was not dose-dependent as the increase was seen only in 100 and 1000 mg/kg groups. Therefore, it is unlikely that *P.campestris* extract resulted in organ-level toxicity in rats.

The hematopoietic system is the common and sensitive target for toxic substances. It serves as a broad indicator of the overall physiological and pathological status of the body [23]. When translating data from animal research, the hematological parameters provide a higher level of predictability of toxicity in humans [15]. All the hematological parameters studied in the present study were within the reference range for rats [24] and the values of treated animals were comparable to control except for an increase in PLT in the 300 mg/kg group. The increased PLT value in the 300 mg/kg treated animals was also within the normal range and was not dose-dependent, hence it was considered as an incidental finding. As a result, the *P.campestris* extract was found to have no harmful effects on the hemopoietic system. However, the present study did not evaluate all hematological parameters including Complete Blood Count (CBC), clotting-related parameters, which must be further studied.

The liver function can be assessed by measuring the levels of protein, bilirubin, and liver enzymes [25]. The elevated levels of AST and ALT are usually associated with liver damage [26]. All the biochemical parameters studied in the present study showed no significant changes except a significant decrease in AST levels in the 300 mg/kg group. The most sensitive marker for hepatocyte damage is ALT, which is found mainly in the liver whereas AST is also found in red blood cells, cardiac and skeletal muscles, and kidneys apart from the liver. Further, the mean values of AST were low at the 300 mg/kg group whereas, at the 1000 mg/kg group, it was increased and levels were comparable with control. So, it can be considered biologically not significant and could be an incidental finding. This statement was further confirmed with normal liver histology in 300 mg/kg treated rats. So, it can be concluded that the *P.campestris* extract is not toxic to the liver.

The increased levels of blood urea nitrogen and creatinine can indicate the impaired kidney function [27]. In the present study, the values of urea, creatinine, sodium and potassium ions, glucose, and other parameters related to kidney function were within the normal range and did not differ significantly in all the animals. So, the *P.campestris* extract did not harm the kidney.

The histopathological examination is used to back up the hematological and biochemical findings [28]. All the organs showed normal architecture comparable to control in the histopathological study. Although some variations were noticed, they were minimal and matched the control group observations, and were not dose-dependent. The lungs showed a thickened alveolar wall with mild congestion in all the groups including control animals, so it was not considered as a treatment-related effect. The results of the histopathological study validate the claim of *P.campestris* extract to be non-toxic.

Based on the above findings, the No Observed Adverse Effect Level (NOAEL) for *P.campestris* extract is considered as 1000 mg/kg/day. According to the FDA [29] guideline, the human equivalent dose (HED) based on the body surface area was computed as 1000 mg/kg divided by 6.2, which is equal to 161 mg/kg body weight in humans, and 16.1 mg/kg body weight in humans is a safe dose of *P.campestris* extract by oral route in humans, recognizing the safety factor of 10 to HED. Therefore, for an average body weight of 60 kg, the safe human dosage is 966 mg (16.1 × 60) or approximately 1 g, which can be consumed orally for less than 28 days without any adverse effects in males.

Additional studies of genotoxicity, carcinogenicity, and teratogenicity [30] are also required to be done as they will strengthen the safety profile of *P.campestris* extract. This limitation, however, is suggested to be the potential course of our future research.

## Conclusions

The acute and 28-day subacute oral toxicity studies of *P.campestris* extract were carried out in the rat model in the present study. The LD_50_ was greater than 2000 mg/kg for rats since the *P.campestris* extract did not reveal any mortality at all tested doses in the acute toxicity study. The 28-day subacute toxicity study did not exhibit any treatment-related adverse effects on the behavior, feed and water intake, relative organs weights, hematological, biochemical parameters, and gross and histopathology of organs in tested doses. However, 28-day oral administration increased the body weight in males. The No Observed Adverse Effect Level (NOAEL) was determined as 1000 mg/kg/day. It is therefore recommended that *P.campestris* extract can be administered safely to men at approximately 1000 mg (or) 1 g /day total dose for a man weighing 60 kg for a brief period of fewer than 28 days.

## Supplementary Information

**Additional file 1:**

## Data Availability

All data are contained and described within the manuscript.
